# A Stepwise Integrative Approach to Managing a Refractory Recurrent Cervical Sialocele in a Dog

**DOI:** 10.3390/ani16020240

**Published:** 2026-01-13

**Authors:** Suhyun Lee, Sang-Kun Jang, Duwhan Park, Hwi-Yool Kim

**Affiliations:** Department of Veterinary Surgery, Konkuk University, Seoul 05029, Republic of Korea; lsh8907@konkuk.ac.kr (S.L.); tkdrns0930@naver.com (S.-K.J.); ppdh888@konkuk.ac.kr (D.P.)

**Keywords:** salivary mucocele, sublingual gland, phenobarbital, OK-432, sclerotherapy, ventral paramedian approach

## Abstract

Recurrent canine cervical sialocele is most often caused by incomplete excision of the mandibular–sublingual gland complex, leading to anatomical distortion and concealment of residual tissue. This case describes the multimodal management of a repeatedly recurrent cervical sialocele in a young, small-breed dog following multiple previous revision surgeries. A stepwise bridging strategy was adopted before definitive salvage surgery. Oral phenobarbital was instituted, resulting in partial reduction in fluid accumulation and improved comfort. Ultrasound-guided intracavitary sclerotherapy with OK-432 was then performed, inducing a localized fibrotic response but without durable cure. Final resolution was achieved only after salvage ventral paramedian (VPM) sialoadenectomy, which provided wide exposure for complete excision of deeply concealed sublingual remnant tissue within a fibrotic pseudocapsule. Histology confirmed a cervical sialocele. Transient neuropraxia resolved within 3 weeks, and no recurrence was observed at 6 months postoperatively. To the authors’ knowledge, this is the first report describing intracavitary OK-432 sclerotherapy as part of a staged multimodal strategy for canine cervical sialocele. This case illustrates the feasibility of integrating medical salivary suppression and minimally invasive sclerotherapy as bridging measures before salvage VPM surgery for refractory cervical sialocele.

## Simple Summary

Salivary cysts (sialoceles) are soft, saliva-filled swellings in dogs. While they are typically cured by surgical removal of the damaged gland, recurrence can occur if a small portion of abnormal tissue is left behind, especially when it becomes hidden within scar tissue from previous surgeries. This report describes the management of a small dog with a repeatedly recurrent neck swelling whose owners were hesitant to proceed directly to another major operation. A stepwise stabilizing plan, adapted from human protocols, was first employed: the anti-seizure medicine phenobarbital reduced saliva production and slowed the rate at which the swelling refilled. Next, a special injectable drug called OK-432 was administered under ultrasound guidance to induce internal scar tissue to make the area firmer and potentially easier to manage at the final surgery. Definitive ventral neck surgery then allowed complete removal of the deeply concealed remaining salivary tissue, and the dog showed no recurrence 6 months later. This case suggests that combining stabilizing medical treatments with a precise, wide surgical approach may provide a long-lasting cure for the most difficult recurrent sialoceles.

## 1. Introduction

Salivary gland diseases are generally uncommon in dogs and cats, with an overall reported prevalence of approximately 0.3% [1]. Among these disorders, sialocele is the most frequently encountered condition in dogs and is defined as a localized accumulation of saliva within the subcutaneous tissues adjacent to a salivary gland or its duct, most commonly arising from the sublingual gland [1,2,3,4,5,6]. Histopathologically, the saliva-filled cavity lacks an epithelial lining and is bordered instead by inflamed connective and granulation tissue; therefore, sialocele is regarded as a saliva extravasation pseudocyst [6]. The etiopathogenesis is frequently unknown, although sialadenitis, trauma, sialolithiasis, foreign bodies, and neoplasia have been reported as possible causes [2,3,5,6,7,8,9,10].

The currently accepted standard treatment for cervical and sublingual sialoceles in dogs is the en bloc removal of the ipsilateral mandibular and sublingual gland–duct complex [2,4,6]. Several surgical approaches have been described, including a combined lateral and intraoral approach, a ventral paramedian (VPM) approach, and a lateral (LAT) cervical approach [6,9,11]. Recurrence after sialoadenectomy is uncommon (≤5%) and is most often associated with incomplete excision of the affected salivary tissues [2,4,6,9]. Conventional conservative management, such as incision or drainage alone, may provide transient relief but carries a high risk of recurrence and is not usually recommended [2,5].

In human head and neck medicine, no universally accepted, evidence-based guidelines exist for the management of salivary gland sialoceles and salivary fistulas [12], and a wide variety of conservative and surgical techniques have been described [13,14,15]. Several authors have proposed stepwise, minimally invasive protocols for chronic salivary leaks and pseudocysts that combine repeated aspiration with pressure dressings [15,16], pharmacologic salivary suppression (e.g., transdermal scopolamine) [17], botulinum toxin chemodenervation [12,18,19], and intracavitary sclerotherapy using various sclerosants, such as OK-432 [13,20,21,22,23]. These approaches are often favored as presurgical alternatives because of their relative economic and cosmetic advantages and reduced risk of iatrogenic nerve injury [13,14,15,21,24]. Veterinary experience with sclerotherapy for salivary lesions, however, remains limited. In dogs, pharmacologic modulation of salivary secretion has also been explored; phenobarbital-responsive salivary disorders, including sialoadenosis and sialadenitis, have been reported in dogs, suggesting a potential neuromodulatory approach to pathological hypersalivation [25,26,27,28].

Therefore, this case report describes the multimodal management of a complex, multiply recurrent cervical sialocele in a dog using both conservative and surgical modalities. Specifically, we report phenobarbital-mediated salivary suppression, minimally invasive OK-432 sclerotherapy, and a VPM salvage sialoadenectomy, and we discuss the potential applicability of a standardized, stepwise, minimally invasive strategy—analogous to that used in human medicine—for refractory salivary gland disease in dogs.

## 2. Case Presentation

A 2-year-old, 4.3-kg, spayed female Maltipoo was referred to the Konkuk University Veterinary Hospital for evaluation of a sixth recurrence of a left cervical sialocele. The dog had a 14-month history of salivary disease that initially presented as bilateral sialoceles with a left ranula and was treated with bilateral mandibular and sublingual sialoadenectomy combined with intraoral marsupialization. Over the subsequent months, the dog underwent multiple additional cervical revision surgeries, including lateral cervical exploratory approaches and ventral approaches, as well as repeated drainage and medication of oral corticosteroids and antibiotics, but recurrence could not be controlled.

**Figure 1:**
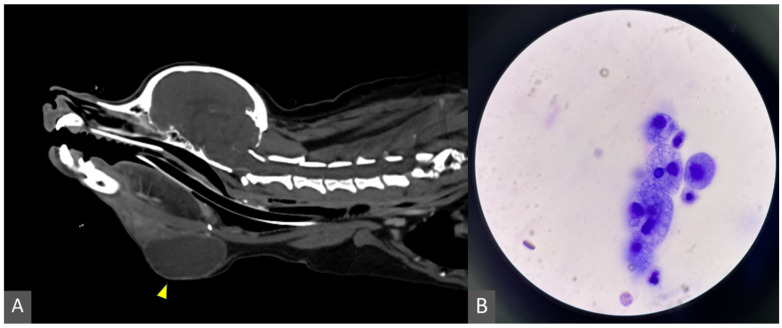
Diagnostic imaging and cytologic findings of the cervical sialocele. (**A**) Contrast-enhanced CT image (sagittal plane) showing a well-circumscribed, fluid-filled mass in the left ventral cervical region, consistent with a cervical sialocele (arrowhead). (**B**) Cytologic examination of aspirated fluid revealing abundant mucoid material and vacuolated macrophages (H&E stain, 1000× magnification).

At presentation, a soft, fluctuant, nonpainful ventral cervical swelling was noted on the left side. Cytological examination was performed on aspirated fluid obtained by fine-needle aspiration, and the material was processed into hematoxylin and eosin (H&E) stained preparations for cytologic evaluation. Cytology revealed extravasated mucoid material with a mixed inflammatory cell population (Figure 1). Aerobic and anaerobic bacterial cultures were performed on the aspirated fluid on multiple occasions, with no bacterial growth detected. Contrast-enhanced computed tomography (CT) was performed using a CT scanner (Aquilion Lightning; Canon Medical Systems, Otawara, Japan) following intravenous administration of iodinated contrast medium (iohexol; Omnipaque, GE Healthcare, Chicago, IL, USA). The CT images demonstrated a well-circumscribed, fluid-filled lesion in the left ventral cervical region, consistent with a cervical sialocele (Figure 1). CT sialography was attempted; however, the contrast medium failed to opacify any residual ductal structures.

### 2.1. Stepwise Conservative Management

Given the refractory nature of the disease, the history of multiple failed surgeries, and the owner’s reluctance to pursue immediate further extensive surgery, a stepwise conservative bridging plan, drawing on human head and neck protocols, was adopted.

#### 2.1.1. Salivary Drive Suppression (Phenobarbital)

Oral phenobarbital (2 mg/kg PO q12h; Hana Phenobarbital Tab., Hana Pharm Co., Ltd., Seoul, Republic of Korea), administered together with hepatoprotective agents, was initiated to address a possible concurrent idiopathic phenobarbital-responsive sialoadenosis (PRS) or sialadenitis. Daily aspirated volume decreased from approximately 3 mL at baseline to about 0.5 mL, and the aspiration interval lengthened (from twice weekly to once every two weeks), indicating partial suppression of salivary output.

However, because of owner compliance issues, serial trough serum phenobarbital concentrations during the first two months remained largely subtherapeutic (approximately 11–19 μg/mL; therapeutic range 20–30 μg/mL). The daily aspirate volume subsequently stabilized at around 1 mL/day. After 58 days, the dose was increased to 3 mg/kg PO, which again reduced the aspirated volume to approximately 0.25–0.5 mL/day for about two months before it gradually plateaued near 1 mL/day; at that time, the trough serum concentration was 23.0 μg/mL, within the lower end of the therapeutic range. Blood samples for serum biochemical analysis and phenobarbital trough concentration measurement were collected by peripheral venipuncture immediately before the next scheduled phenobarbital dose and processed using standard clinical laboratory methods. Serial serum biochemical profiles, including alanine aminotransferase (ALT) and alkaline phosphatase (ALP) activities, remained within reference intervals throughout phenobarbital therapy (Table 1). Although the owner was highly satisfied with the clinical improvement, the persistent need for intermittent drainage and the potential risks associated with long-term phenobarbital therapy indicated that further local intervention was required. Sclerotherapy was therefore planned as the next step before considering surgical management of the sialocele.

**Figure 2:**
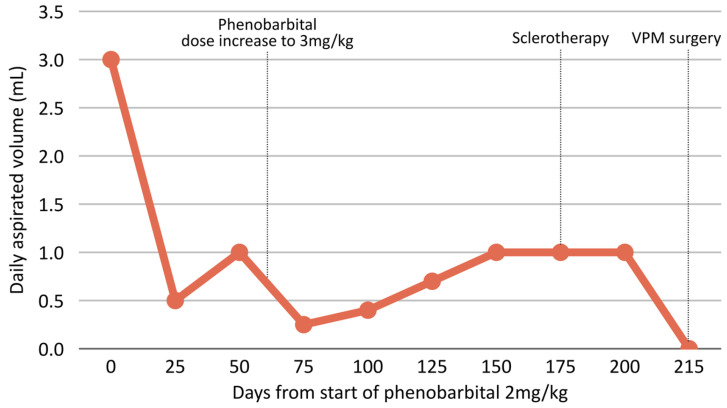
Estimated daily aspirated fluid volume (mL) from the sialocele over time in relation to key interventions. The curve shows an initial decrease, then stabilization around 1 mL/day. Vertical dashed lines indicate phenobarbital dose escalation to 3 mg/kg (day 58), OK-432 sclerotherapy (day 178), and salvage VPM sialoadenectomy (day 214), after which no further fluid accumulation was detected.

The overall trend in estimated daily aspirated volume during phenobarbital therapy and subsequent interventions is summarized in Figure 2.

#### 2.1.2. Percutaneous Sclerotherapy (OK-432)

Ultrasonographic examinations were performed and interpreted by two experienced veterinarians (a board-certified radiologist and a senior surgery resident) using a high-resolution ultrasound system (V8; Samsung Medison, Seoul, Republic of Korea). Sclerotherapy was performed under ultrasonographic guidance, with the dog in deep sedation achieved using medetomidine hydrochloride (20 µg/kg IV; Domitor, Zoetis, Parsippany, NJ, USA) and midazolam (0.2 mg/kg IV; Bukwang Midazolam Inj., Bukwang Pharm, Seoul, Republic of Korea). After routine clipping and aseptic preparation, OK-432 (Picibanil, Chugai Pharmaceutical, Tokyo, Japan) was reconstituted in 10 mL of sterile normal saline (0.1 Klinische Einheit [KE] per milliliter; 0.01 mg/mL). A 21-gauge catheter was advanced percutaneously into the cyst lumen under ultrasound guidance. Once the catheter tip was confirmed within the cavity, as much fluid as possible was aspirated and the aspirated volume recorded. The syringe was then exchanged for one containing the OK-432 solution, and a volume of sclerosant equal to the volume of fluid removed was slowly injected into the cavity, avoiding extracystic leakage. The catheter was removed and medetomidine was reversed with atipamezole (50 µg/kg IM; Antisedan, Zoetis, Parsippany, NJ, USA). Firm digital pressure was then applied for approximately 10 min, followed by a compressing dressing for about 5 h.

Post-procedural analgesia included meloxicam (0.2 mg/kg SC; Metacam, Boehringer Ingelheim Vetmedica, Ingelheim, Germany), butorphanol tartrate (0.2 mg/kg IV; Butorphan inj., Myungmoon Pharm Co., Ltd., Seoul, Republic of Korea), and gabapentin (20 mg/kg PO q12h; Gabapentin 100 mg capsule, Dong-A ST, Seoul, Republic of Korea). The dog was monitored overnight and was discharged the following day with continued oral analgesic therapy.

**Figure 3:**
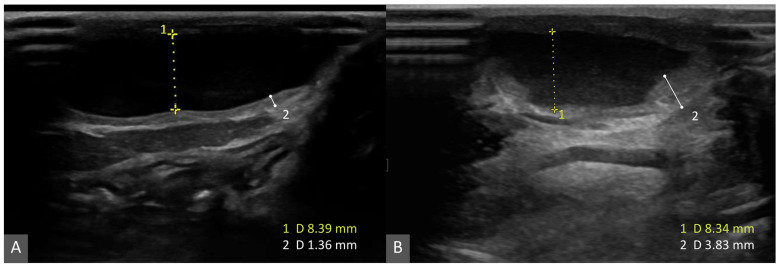
Longitudinal ultrasonographic images of the cervical sialocele. (**A**) Baseline image before sclerotherapy. (**B**) Follow-up image after sclerotherapy. The dotted calipers (1) indicate maximal cyst depth ((**A**) 8.39 mm; (**B**) 8.34 mm), and the solid white calipers (2) indicate cyst wall/septal thickness ((**A**) 1.36 mm; (**B**) 3.83 mm). Cyst depth is similar between time points, whereas the cyst wall/septum is thicker after sclerotherapy, consistent with a localized inflammatory and fibrotic response.

Minor complications included localized erythema at the injection site, transient low-grade fever (rectal temperature up to 39.8 °C), and pain on palpation; all resolved within 3 days. Local hyperpigmentation was also noted at the injection site. Transient elevation of C-reactive protein (CRP) and ALP were observed but returned to within 7 days. Follow-up ultrasonography demonstrated cyst wall thickening, consistent with a localized inflammatory and fibrotic response (Figure 3). Fluid aspirated after the procedure showed transient hemorrhagic change but reverted to a typical mucoid appearance within 2 weeks.

Despite these changes, the procedure was non-curative, and gradual reaccumulation of fluid was observed thereafter. Phenobarbital therapy was continued at the same dosage, and the estimated rate of fluid reaccumulation, based on serial aspirations, remained approximately 1 mL/day, similar to the stabilized rate observed before sclerotherapy.

### 2.2. Definitive Salvage Surgery

On the day of surgery, the dog was premedicated with midazolam (0.2 mg/kg IV) and fentanyl citrate (2 µg/kg IV, then 1–4 µg/kg/h IV continuous rate infusion (CRI) intraoperatively; Fentanyl Citrate Inj., Hana Pharm, Seoul, Republic of Korea). Anesthesia was induced with propofol (4 mg/kg IV; Anepol Inj., Hana Pharm, Seoul, Republic of Korea) and maintained with isoflurane (Terrell Solution, Kyongbo Pharm, Asan, Republic of Korea) in oxygen. Prophylactic antibiotic treatment with amoxicillin–clavulanate (13.75 mg/kg IV; Amocla, Kuhnil Pharmaceutical, Seoul, Republic of Korea) was administered 30 min before surgery and repeated every 90 min intraoperatively.

**Figure 4:**
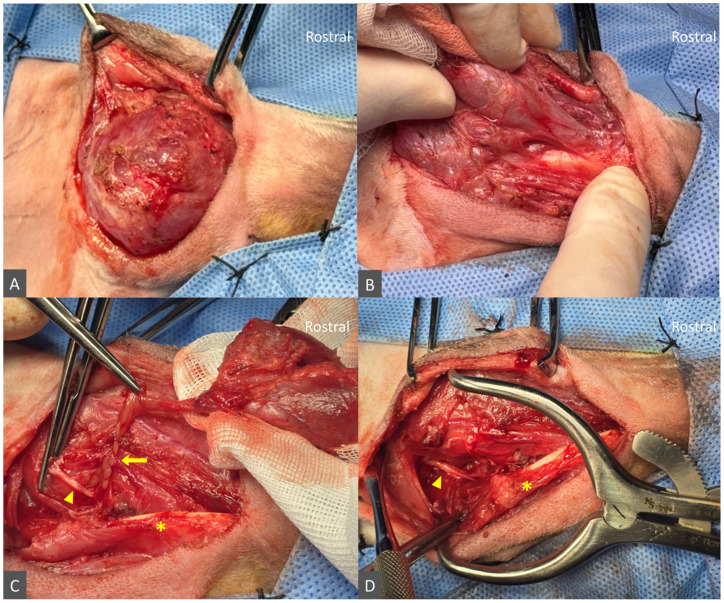
Salvage VPM sialoadenectomy for recurrent cervical sialocele. (**A**) Ventral paramedian approach exposing a large, thick-walled cystic mass with a fibrotic pseudocapsule. (**B**) Circumferential dissection of the pseudocapsule with gentle traction to expose deeper structures. (**C**) Deeper dissection showing the residual mandibular–sublingual duct and rostral sublingual tissue (arrow) beneath the partially incised mylohyoideus muscle (asterisk), with the hypoglossal nerve (arrowhead) and exposed mandibular body visible dorsally. (**D**) Surgical bed after en bloc excision of the gland–duct–pseudocapsule complex.

The dog was positioned in dorsal recumbency with the neck extended and rotated contralaterally to maximize exposure of the left ventral cervical region. The surgical site was clipped and aseptically prepared. A ventral paramedian skin incision was made in the intermandibular region over the cervical swelling. The subcutaneous tissues and platysma muscle were incised and elevated, revealing a large, thick-walled, cystic structure with a well-developed fibrous capsule and dense adhesions. Using a combination of sharp and blunt dissection, the pseudocapsule and surrounding adhesions were dissected circumferentially, following the lesion margin to avoid rupture or further leakage. Gentle traction on the lesion allowed progressive exposure of the residual sublingual gland–duct complex deep to the digastricus muscle and along the medial aspect of the mandible. As the residual duct and associated sublingual gland tissue were traced rostrally and deeper toward the mandible, a partial incision of the mylohyoideus muscle was required to achieve complete excision of all remnant tissue. The duct was ligated as far rostrally as practicable with 4-0 polydioxanone (PDS) and transected, and the remaining fibrotic capsule, duct, and associated sublingual gland tissue were excised en bloc (Figure 4).

Grossly, the excised sublingual sialocele and duct complex measured approximately 63.5 × 50.8 mm. Recovery from anesthesia was uneventful. Postoperative analgesia included a fentanyl CRI, meloxicam and gabapentin.

### 2.3. Histopathology

**Figure 5:**
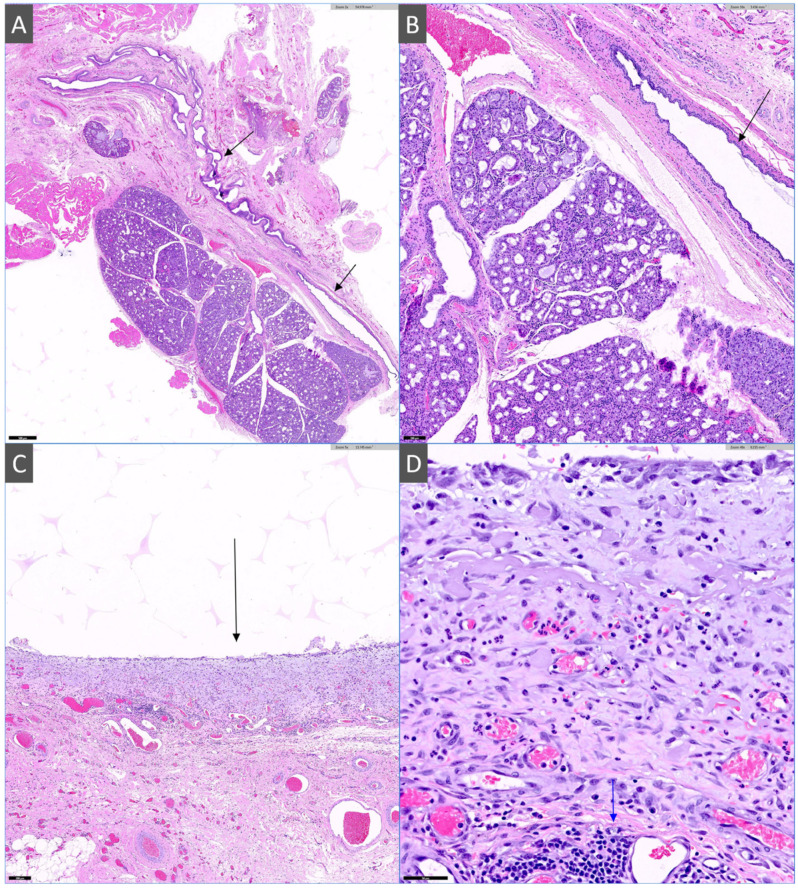
Histologic features of the cervical sialocele and associated salivary gland (H&E stain). (**A**) Salivary gland parenchyma and ectatic salivary duct (arrows), 2× magnification (**B**) Higher magnification of the ectatic duct (arrow) lined by attenuated epithelium and surrounded by fibrous connective tissue, 10× magnification (**C**) Pseudocyst inner wall (arrow) composed of dense fibrous connective tissue consistent with a mature fibrotic pseudocapsule, 5× magnification. (**D**) High-power view of the pseudocyst wall and adjacent salivary tissue showing lymphoplasmacytic inflammation (arrow), 40× magnification.

Histopathological examination was performed on formalin-fixed, paraffin-embedded tissue sections stained with H&E and evaluated by a board-certified veterinary pathologist. Examination revealed a periglandular pseudocyst (sialocele) with ductal ectasia. The pseudocyst consisted of a moderately sized cavity that was partially lined by attenuated epithelium, with extensive areas of epithelial loss. It was surrounded by a moderately thick rim of compressed, mature fibrous connective tissue infiltrated by lymphocytes, plasma cells, and histiocytes, consistent with a well-formed fibrotic pseudocapsule. Within the adjacent salivary tissue, there were diffuse, mild-to-moderate, chronic lymphoplasmacytic sialadenitis and mild interstitial fibrosis (Figure 5).

Taken together with clinical history, these findings are compatible with a chronic cervical sialocele associated with a chronic inflammatory component and mature fibrous pseudocapsule formation.

### 2.4. Clinical Course and Outcome

**Figure 6:**
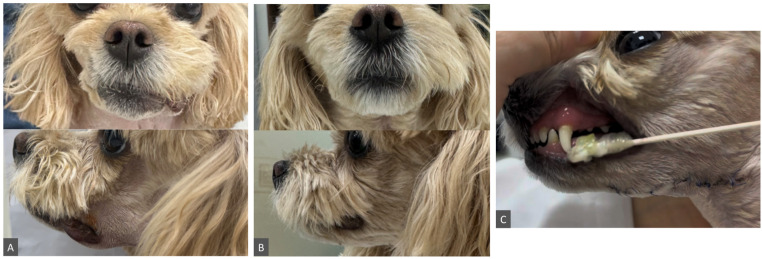
Clinical manifestations of transient neuropraxia after surgery. (**A**) POD 3, showing mild unilateral lip drooping and drooling (top, frontal view; bottom, lateral view). (**B**) POD 23, demonstrating complete resolution with symmetrical facial expression and no evidence of drooling or lip drooping. (**C**) Tongue dysfunction manifested by accumulation of saliva and food in the buccal mucosa.

The challenging VPM dissection achieved complete anatomical clearance of the residual mandibular–sublingual gland–duct complex. Postoperative complications were limited to mild surgical site swelling, which resolved within 1 week, and transient neuropraxia, most likely involving branches of the facial and hypoglossal nerves based on the observed clinical signs. The latter manifested as drooling, mild lip drooping, and tongue dysfunction characterized by accumulation of saliva and food in the buccal mucosa. These signs resolved spontaneously by postoperative day (POD) 23 (Figure 6). At the 6-month follow-up, the cervical swelling had completely resolved with no evidence of recurrence, supporting the VPM approach as the definitive salvage procedure in this dog.

## 3. Discussion

This case illustrates how a stepwise, minimally invasive strategy, adapted from human head and neck protocols, can be integrated with salvage VPM sialoadenectomy to manage a repeatedly recurrent cervical sialocele in a dog, rather than relying solely on repeated conventional surgery.

Recurrent cervical sialoceles are most commonly attributed to incomplete removal of the mandibular–sublingual gland–duct chain, particularly residual rostral polystomatic lobules or ductal remnants [5,6,9,29]. Leakage from more rostral portions of the sublingual chain typically presents as a ranula, whereas more caudal leakage points tend to produce a cervical sialocele [5,29]. With repeated revision surgery, progressive fibrosis, distortion of fascial planes, and caudoventral migration of residual glandular tissue can further displace remnant tissue into deeper cervical soft tissues, placing it beyond the practical reach of a standard LAT or intraoral approach [9,10,29]. However, recent anatomical evidence suggests a more nuanced mechanism. Solitary lobules of the monostomatic sublingual gland are frequently distributed along the course of the major sublingual duct, often in deep or rostral positions relative to the primary gland body [30]. Such concealed lobules may be overlooked during conventional lateral approaches, providing a plausible explanation for postoperative recurrence even when the main gland body appears to have been adequately excised [30].

Accordingly, the VPM approach is preferred as a salvage option because it provides wide ventral exposure along the mandibular–sublingual chain, facilitating removal of deeply displaced remnant tissue despite a higher incidence of wound-related complications [9,10]. In this dog, multiple prior explorations failed to achieve durable control, and long-term resolution was obtained only after salvage VPM sialoadenectomy, underscoring the importance of wide, deep exposure when remnant tissue is concealed by fibrosis and caudoventral displacement.

The role of pseudocapsule excision in sialocele surgery remains unclear [10]. In one retrospective series, recurrence was unrelated to pseudocapsule removal, suggesting that meticulous excision of the mandibular–sublingual gland–duct complex is more important than routine pseudocapsule resection for preventing relapse [10]. Nevertheless, in this recurrent, fibrotic case, the thickened pseudocapsule served as a guide during VPM exploration to track the displaced residual glandular and ductal tissue, permitting proximal dissection toward the leakage source within the deep cervical fascial planes.

Beyond definitive excision, this case highlights the potential value of staged medical and minimally invasive interventions to stabilize refractory cervical sialoceles. This strategy parallels human head and neck protocols, in which chronic salivary leaks and pseudocysts are managed initially with salivary suppression and intracavitary sclerotherapy [12,15,19,21]. In this case, phenobarbital and OK-432 were used as bridging measures to reduce salivary inflow and consolidate the pseudocyst before salvage surgery.

Phenobarbital-responsive salivary disorders (e.g., PRS), described only in dogs, typically present with bilateral salivary gland enlargement, hypersalivation, retching, and vomiting, showing marked improvement after treatment [25,26,27,28]. Although the pathophysiology remains incompletely understood, a limbic epileptic mechanism with neuromodulatory effects on central autonomic pathways controlling salivary flow has been proposed, likely involving GABA-A–mediated modulation of glandular secretion or ductal smooth muscle tone [25,26]. Given the dog’s bilateral involvement and multiply recurrent course, phenobarbital was introduced as a therapeutic trial targeting putative neurogenic and inflammatory drivers of pathological salivary secretion.

After phenobarbital was initiated, the rate of sialocele reaccumulation decreased, with lengthened intervals between aspirations and reduced aspirated volumes, consistent with a neuromodulatory antisialagogue effect. While the effect persisted, it ultimately plateaued, and the sialocele did not resolve completely. Continued reliance on intermittent drainage and the unresolved structural source of leakage carried an ongoing risk of infection. Although clinically well tolerated in this case, the recognized risks of phenobarbital (e.g., sedation, hepatotoxicity) still warrant regular biochemical monitoring [31]. The partial response may reflect either a concurrent phenobarbital-responsive salivary disorder or non-specific central effects on salivary flow. Functionally, suppression of salivary drive limited ongoing tissue autolysis and allowed the cavity to stabilize rather than rapidly re-expand [17], but the underlying mechanical leak persisted. Thus, in this context, phenobarbital can be regarded as a stabilizing adjunct that provides meaningful yet incomplete functional modulation, rather than a definitive treatment for cervical sialocele.

Because phenobarbital and drainage alone did not provide definitive control, OK-432 (Picibanil) was selected as a translational sclerosing agent to provide a minimally invasive local intervention. OK-432, a lyophilized *Streptococcus pyogenes* preparation, induces a localized inflammatory and immunomodulatory response upon intracavitary injection, leading to subsequent fibrosis and obliteration of the cyst cavity [32]. Intracavitary sclerotherapy using sclerosants such as ethanol, OK-432, sodium tetradecyl sulfate (STS), or bleomycin/fibrin-glue combinations, has been successfully applied to sialoceles and related salivary pseudocysts in humans [13,14,20,21,22,23,24,33]. Among these agents, OK-432 is particularly well-established as a first-line treatment for macrocystic lymphatic malformations and has also shown efficacy for intraoral and plunging ranulas and postoperative parotid sialoceles [13,20,21,22,23,24]. Plunging ranula is considered conceptually analogous to canine cervical sialocele, as both represent salivary extravasation pseudocysts arising from the sublingual gland and extending into the cervical soft tissues [13].

In human series, OK-432 is typically reconstituted at 1 KE in 10 mL of normal saline and injected in a volume equivalent to the aspirated cyst content, a dosing scheme that was adopted in the present case [21,22,23]. Ultrasound-guided injections are usually repeated at several-week intervals until the lesion collapses. Success rates approach 100% when multiple sessions are administered, whereas single-session success is considerably lower, reported at 45.5% and 33.3% in two clinical series of plunging ranulas [13,23].

In this dog, a single OK-432 injection did not achieve durable resolution. Although the lesion became firmer with ultrasonographic evidence of cyst wall thickening—findings comparable to the localized fibrotic response reported in human series [21,34]—overall size and fluid reaccumulation were only minimally affected, consistent with the limited efficacy reported for single-session therapy [13,23]. Histopathology was consistent with a chronic inflammatory pseudocyst with mature fibrous encapsulation, supporting the long-standing nature of the lesion but not allowing distinction between treatment-related change and disease chronicity [3].

The complication rate of OK-432 sclerotherapy has generally been reported as low [13,22,23], and a similar profile was observed in this case: transient local pain, swelling, and low-grade fever resolved within 3 days, and no serious systemic or neurologic adverse effects were noted. Mild skin discoloration developed at the injection site but was considered cosmetic only. To the authors’ knowledge, this is the first description of intracavitary OK-432 sclerotherapy for a salivary sialocele in a dog, supporting its potential role as a bridging intervention rather than a stand-alone curative treatment.

Taken together, phenobarbital and OK-432 did not independently resolve the disease but plausibly functioned as bridging measures that reduced salivary inflow and promoted cavity consolidation before the final salvage procedure. Conceptually, sialoadenectomy removes the primary salivary source, whereas sclerotherapy targets the extravasation cavity and its immediate surroundings, and complementary use of these modalities has been proposed in a previous human head and neck report [33]. By analogy, we hypothesize that OK-432–induced fibrosis contributed to a firmer, more clearly delineated lesion, potentially facilitating complete excision during salvage VPM approach. However, this complementary interaction is regarded as hypothesis-generating rather than definitive.

From a safety standpoint, the multimodal strategy used here appeared acceptable. The VPM approach entails extensive dissection and has been associated with a higher rate of wound-related complications (e.g., swelling, seroma, dehiscence) than LAT approach [9,10]. Tunnelization of the mandibular–sublingual chain dorsal to the digastricus muscle requires a wider field of dissection, theoretically increasing the risk of injury to adjacent structures (e.g., hyoid apparatus, hypoglossal nerve, internal carotid artery) [10]. Consistent with this potential risk, transient neuropraxia (manifested by drooling, lip drooping, and tongue dysfunction) was observed postoperatively. However, these reversible deficits resolved completely within 3 weeks. Such outcomes are generally considered an acceptable trade-off for durable cure in recurrent disease.

This report has several limitations. As a single case without a control group, it is not possible to clearly distinguish the individual contributions of the multimodal regimen—phenobarbital, OK-432 sclerotherapy, and definitive VPM sialoadenectomy—nor to make strong causal inferences regarding any apparent adjunctive effects. Furthermore, the administration of phenobarbital had an insufficient evidence base, and the patient’s presentation differed from previously reported responsive disorders, making the appropriateness and generalizability of this approach uncertain. Regarding OK-432, it was administered only once, whereas human protocols typically employ repeated, imaging-guided injections; consequently, evidence-based guidelines for sclerotherapy in canine sialoceles (including agent selection, dose, volume, and retreatment intervals) have not yet been established. The suggestion that sclerotherapy-induced fibrosis may have helped delineate residual glandular tissue should therefore be regarded as speculative. Although the results of the present case are promising, they are not sufficiently conclusive to establish this protocol as a standard treatment. Prospective, multi-case studies with standardized medical and sclerotherapy protocols and extended follow-up are required before routine clinical application of this multimodal approach for managing refractory salivary gland disease in dogs can be considered.

## 4. Conclusions

This case report demonstrates the feasibility of integrating a stepwise, multimodal strategy, adapted from human head and neck protocols, for the management of a multiply recurrent cervical sialocele. The minimally invasive measures—specifically phenobarbital-mediated salivary suppression and intracavitary OK-432 sclerotherapy—did not achieve a stand-alone cure, but they functioned as effective bridging interventions to reduce salivary inflow and consolidate the pseudocyst prior to definitive salvage surgery. Crucially, following multiple prior unsuccessful revision procedures, VPM sialoadenectomy achieved durable resolution. This success underscores the necessity of definitive surgical management for refractory recurrent sialocele and the importance of wide ventral exposure and meticulous dissection to remove migrated mandibular–sublingual gland–duct remnants in anatomically distorted recurrences. The present findings illustrate a potential management option rather than a definitive clinical standard and should be interpreted with caution. Nevertheless, this case supports further prospective evaluation of a stepwise, multimodal approach to better define its long-term efficacy and safety in dogs with refractory salivary gland disease.

## Figures and Tables

**Table 1:** Phenobarbital dosing, trough serum concentrations, and selected serum biochemical parameters during conservative management.

Parameter	Day 11	Day 38	Day 58	Day 98	Day 198	Reference Range
Phenobarbital dose (mg/kg PO q12h)	2.0	2.0	3.0 ^†^	3.0	3.0	-
Trough phenobarbital concentration (µg/mL)	14.7	11.3	16.0	18.9	23.0	20–30
ALT (U/L)	51	24	29	30	28	10–125
ALP (U/L)	57	91	105	115	171	23–212

^†^ Phenobarbital dose was increased to 3 mg/kg PO q12h on Day 58.

## Data Availability

The authors declare that the data found in this paper are accessible and available.

## Abbreviations

The following abbreviations are used in this manuscript:

VPM	Ventral paramedian
LAT	Lateral approach
KE	Klinische Einheit
PRS	Phenobarbital-responsive sialoadenosis
POD	Postoperative day
CRI	Continuous rate infusion
H&E	Hematoxylin and eosin
